# A challenging interplay between basic research, technologies and medical education to provide therapies based on disease mechanisms

**DOI:** 10.3389/fmed.2024.1464672

**Published:** 2024-08-20

**Authors:** Victoria I. Bunik

**Affiliations:** ^1^Belozersky Institute of Physicochemical Biology, Lomonosov Moscow State University, Moscow, Russia; ^2^Faculty of Bioengineering and Bioinformatics, Lomonosov Moscow State University, Moscow, Russia; ^3^Department of Biochemistry, Sechenov University, Moscow, Russia

**Keywords:** Charcot-Marie-Tooth neuropathy, deep mutational scanning, disease phenotype, gene and protein function, genotype-phenotype relationship, next generation sequencing, pathogenic amino acid substitutions, pyruvate dehydrogenase

Identification of disease-causing genes and understanding disease mechanisms are critical challenges in the translational and precision, or personal, medicine. Principal problems in addressing these challenges are illustrated in Figure 1. On one hand, many pathogenic factors, including mutations of causative genes, converge to manifest the same disease symptoms. On the other hand, a disease may have different phenotypes even when the same gene is damaged. Complexity of biological functions also plays a role in the genotype-phenotype relationship. Specific metabolic impairments, especially in central metabolism, often have prominent metabolic markers for a rather straightforward identification. The more complex the function, the more causative genes and different pathways may contribute to its impairment, leading to permanently extending lists of such genes. Examples of such more and less obvious genetic origins and phenotypes of diseases are considered below along with new technologies contributing to deciphering the genotype-phenotype relationship on the cross-roads of basic research and medicine.

**Figure 1:**
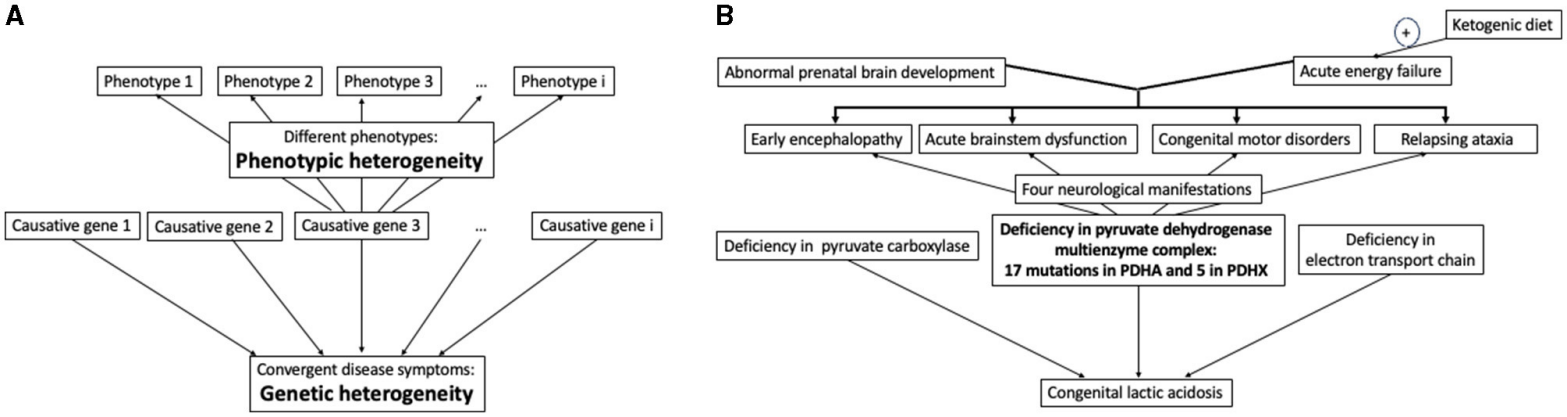
Relationship between the disease causing genes and phenotypes. **(A)** In general, defects in different genes may converge to the same symptoms, with multiple disease phenotypes potentially observed at even the same defect of one gene. **(B)** Illustration of the relationship in **(A)** by specific case of congenital lactic acidosis and its phenotypes caused by impairments of the pyruvate dehydrogenase multienzyme complex due to defective genes PDHA and PDHX, encoding for the α-subunit of the pyruvate dehydrogenase and protein X, correspondingly. See text for further explanations.

Different types of organic aciduria usually point to a limited number of metabolic pathways whose defect enzymes or transporters must be examined. Even in these relatively simple cases, exemplified in Figure 1B by congenital lactic acidosis, additional complexity arises when the impairment affects a metabolic branch point. Under this condition the number of potentially involved metabolic and regulatory proteins is increased. For instance, impairments of different enzymes participating in or linked to aerobic oxidation of glucose—an indispensable energy substrate for the brain—result in congenital lactic acidosis with serious neurologic manifestations (Figure 1B). The most common reason of this state, however, is deficient pyruvate dehydrogenase reaction (1), an essential branch point that couples glycolysis with mitochondrial oxidative metabolism. The physiologically relevant pyruvate dehydrogenase reaction in humans is catalyzed by a multienzyme complex comprising multiple copies of the three enzymes and additional protein (protein X) binding the terminal enzyme component. These complex-forming proteins are encoded by five different genes, two of them coding for the alfa and beta subunits of the pyruvate dehydrogenase heterotetramer. Therefore, the overall pyruvate dehydrogenase reaction is affected by pathogenic mutations in these five genes for the component enzymes (1–3). A study of 22 molecular defects in the two protein components of the multienzyme complex of pyruvate dehydrogenase has revealed four types of neurological manifestations with the two major pathological signs (Figure 1B). Overall, phenotypes of the permanently extending list of diverse mutations in the pyruvate dehydrogenase complex range from fatal lactic acidosis in newborns to chronic neurological dysfunction. The pyruvate dehydrogenase reaction may also be impaired by mutations in the regulatory enzymes, determining (de)phosphorylation of the pyruvate dehydrogenase complex, i.e., the pyruvate dehydrogenase kinases and phosphatases. For instance, demyelinating polyneuropathies may be induced by certain mutations of the pyruvate dehydrogenase alfa subunit (gene PDHA) or in an isoenzyme of pyruvate dehydrogenase kinase (gene PDK3) (4, 5), all inactivating the pyruvate dehydrogenase reaction. The known mutations in the genes encoding the catalytic and regulatory components of pyruvate dehydrogenase complex, have different impact on the enzyme activity, and the *in vitro* activity levels in the multienzyme complex with different mutations do not correlate with patients' phenotypes (3, 6, 7). Worth noting in this regard that the *in vivo* levels of enzymatic activities depend on supramolecular complexes and specific saturations with substrates and coenzymes, which cannot be modeled upon *in vitro* assays of the enzymes. This difference may contribute to poor correlation between the *in vitro* levels of activities inherent in the mutated enzymes, and disease phenotypes. According to some studies, most of substitutions in proteins affect their interactions rather than stability or activity, and phenotype may greatly depend on the mutation-changed protein-protein interactions (8, 9). In case of multienzyme complexes, such as that of pyruvate dehydrogenase, the changed interaction between components of the complex could affect the overall activity of the complex assayed *in vitro* (9). However, perturbed formation of supramolecular structures of a higher level, such as, e.g., incorporation of the pyruvate dehydrogenase complex into the tricarboxylic acid cycle metabolon, would not necessarily affect the *in vitro* assay of pyruvate dehydrogenase activity, yet detrimental phenotype may arise due to impaired substrate channeling in the metabolon. Other factors, potentially perturbing the correlation between the activity of mutated enzyme and phenotype of a patient, may include the time of diagnosis, chosen treatment and time of the treatment start, as these factors may cause different disease outcomes even at identical mutations in siblings (10). Thus, genetic screening should be complemented by the enzymatic assays *in vitro*, but cannot be substituted by the assays.

As noted above, congenital lactic acidosis illustrates a “simple” case for finding the genotype-phenotype relationship as (i) the perturbation occurs in central metabolism of glucose, and (ii) mutations-perturbed enzymes are by now well characterized regarding their structure-function relationship. The ensuing understanding of molecular mechanisms underlying specific impairments, greatly helps not only to interprete pathogenicity of new mutations causing the disease condition, but also to elaborate appropriate therapies. In particular, the list of mutations-caused impairments of the pyruvate dehydrogenase reaction is permanently growing. Each case study contributes to a better understanding of not only the genotype-phenotype relationship, but also appropriate therapies. Upon defects of pyruvate dehydrogenase, certain nutritional supplements and ketogenic diet show beneficial effects (1, 3, 7, 11, 12). These actions are in good accord with molecular mechanisms characterized in the *in vitro* studies of the complex. The substrate pyruvate and/or the coenzyme thiamine diphosphate may increase the pyruvate dehydrogenase activity due to the known structural stabilization of the multienzyme complex by the ligands and inhibition of the pyruvate dehydrogenase kinase, the enzyme inactivating pyruvate dehydrogenase by phosphorylation. Benefits of supplementations with arginine and aspartate may be related to positive effects on the amino acid metabolism which is perturbed when pyruvate dehydrogenase reaction is inhibited *in vivo* (13). A long-known and rather efficient approach to relieve the consequences of impaired pyruvate dehydrogenase reaction is ketogenic diet. The diet replenishes the intermediates of the tricarboxylic acid cycle, which are decreased due to insufficient provision of acetyl-CoA to the cycle by dysfunctional pyruvate dehydrogenase complex. The diet is not efficient upon the mutations of pyruvate dehydrogenase leading to prenatal brain malformation, which are often lethal *in utero* (14), but provides improved neurologic outcome and longevity upon acute energy failures due to disorders of the pyruvate dehydrogenase complex (Figure 1B), especially when introduced as soon as possible (7, 10, 15). Remarkably, ketogenic diet is contraindicated up to lethal outcome, if lactic acidosis is due to deficiencies of pyruvate carboxylase or electron transport chain (10). These findings stress importance of knowledge on specific molecular mechanisms for prescription of appropriate treatments.

Severe neurological outcomes of impairments in the pyruvate dehydrogenase reaction demonstrate the highest sensitivity of neural system to defects of glucose metabolism. Besides, the most complex functions, performed by neural system, are simultaneously the most fragile ones. The complex background of neurological impairments is exemplified by very different genetic origins of, e.g., perturbed neurotransmission upon epileptic seizures (16), or hereditary motor-sensory polyneuropathies (12, 17, 18). A multitude of factors, supporting the neural system function, often include poorly characterized proteins whose functions and their impairments are not easily assessed *in vitro*. Besides, manifestation of the impairments as a disease may be delayed in time, or triggered by environmental factors, such as infections or trauma, further complicating our deciphering of relationships between genotype and phenotype. Especially for such complex cases, identification of the disease-causing genes and their functional significance is not straightforward. Our understanding of genotype-phenotype relationships in such cases is greatly advanced by technological developments in high-throughput analysis combined with bioinformatics.

Next generation sequencing (NGS) of DNA and RNA identifies genetic variants in even whole genomes within a short time (19). The technology employs multiple parallel sequencing of the proband DNA fragments, followed by bioinformatic comparison of the proband genome with the reference human genomes. The identified variants of the genes in individual genomes accumulate the knowledge to be translated to clinical applications, such as diagnosis, prognosis and treatment of diseases. In particular, this knowledge is used to develop the disease-specific gene panels for the targeted NGS, that further decreases the screening costs. Based on the available understanding of the genotype-phenotype relationship in certain diseases, such panels help individual diagnostics in precision medicine. For instance, many of genetically caused polyneuropathies or neuromuscular disorders manifest as Charcot-Marie-Tooth disease (CMT), that may be caused by a mutation in a 100 of different genes (17, 18). The phenotypes may be very different even in the same family. A retrospective screening of 55 persons manifesting hereditary muscular and sensory neuropathy used a panel of 55 genes known to be affected in CMT (17). Some cases were confirmed to carry on the pathogenic variants of the known CMT causative genes. However, in other cases the variant pathogenicity remained unclear. The study indicates that many substitutions in important proteins still require characterization in terms of their structure-function relationships to better understand the genotype-phenotype relationships.

Public databases are indispensable for advances in our understanding of the complex disease mechanisms, as the data deposited in such databases allow researchers to reveal associations of genomic substitutions with specific disorders. An example is provided by a bioinformatics study of association between mutations in nuclear genes encoding mitochondrial proteins, and undiagnosed developmental disorders or autism (20). In this study, genomic and transcriptomic data of large (*n* ~ 10^5^) samples of the affected persons and controls are analyzed, presuming pathogenicity of truncated proteins and deleterious missense mutations. Enrichment of these variants in the disorder vs. control samples is identified, while synonymous (presumably non-pathogenic) amino acids substitution are not differently enriched in the same samples. Using the freely available bioinformatics resources, the protein-protein interactions and biological processes of the disorders-enriched proteins are identified, with the disease-enriched genes found to be preferentially expressed in neurons. Gene co-expression analysis indicates that the genes enriched in the undiagnosed disorders and in autism are characterized by specific distribution in different co-expression modules. The finding helps to discriminate genetic causes of autism and those of other analyzed disorders. The disease genes are further prioritized according to the scores of the system intolerance to the mutations. Some of the genes, revealed as significant in this analysis of large samples, are also known to contribute to the considered disorders from independent studies of specific proteins in smaller samples. The consistent results of independent studies heighten potential significance of other prioritized associations (total 130 genes), established in the bioinformatics analysis of the database-deposited variants (20).

Remarkably, top 20 nuclear genes for mitochondrial proteins, significant for the developmental disorders and autism, include alfa-subunit of pyruvate dehydrogenase at the fourth position of the list (20). This finding reciprocates independent observations that deficient mitochondrial metabolism, known to be associated with neurological and cognitive diseases, most often is linked to genetic disorders of pyruvate oxidation (1). As disturbed homeostasis usually compromises cellular capacity to scavenge reactive oxygen species, a vicious cycle of permanently accumulating damage is initiated by dysregulation of pyruvate oxidation. Eventually, different neurological phenotypes with a common marker, such as lactic acidosis (Figure 1B), may arise due to interaction of the original pyruvate oxidation defect with other, genetic or environmental, factors. Hence, in addition to measures combating original defects, the compounds that are able to regulate multiple targets of basic homeostatic significance, attract increasing attention regarding their therapeutic potential in the pathologies progress. An example of natural multi-target compound is vitamin B1 (thiamine). This well-known regulator of glucose metabolism activates pyruvate oxidation and antioxidant defense through its action on multiple targets, the most known being mitochondrial dehydrogenases of 2-oxoacids and cytosolic transketolase. Accordingly, benefits of supplementation with vitamin B1 and its pharmacological forms have been observed in a wide spectrum of neurological disorders of different etiology (21, 22).

Specific studies of potentially disease-associated variants of a protein, carefully selected according to genetic, bioinformatics and structural considerations, and accompanied by clinical phenotype descriptions, are not only helpful for the disease management, but also of conceptual significance (23). However, increasing number of gene variants of unknown significance, generated by NGS, requires high-throughput technologies to assess biological manifestations of these variants. Deep mutational scanning (DMS) is developed to generate the multitude of possible gene variants and assess their effects on living systems (24). The library of gene variants is created and introduced in an appropriate human cell line. Biological significance of the variants is evaluated in a high-throughput assay. Different assay types may consider available knowledge on specific involvement of the studied genetic variant into (i) DNA metabolism (assays of cellular viability addressing changes in DNA replication, repair or modification), (ii) RNA metabolism (reporter constructs for dysregulated transcription and alternative splicing, or mislocalization), (iii) protein metabolism (reporter constructs to estimate protein abundance, protein modification, or localization), (iv) overall metabolism (enzyme- or metabolite-specific assays), and (v) ion fluxes (cytotoxicity assays and bioreceptor-based reporters).

Certainly, the study of protein variants in a cell line cannot substitute clinical descriptions of disease phenotypes in human organism, neither characterize the developmental aspects of this phenotype. However, DMS contributes to the needed understanding on pathogenic significance of a gene variant in a complex cellular milieu. This is very important for filling in the current gaps in understanding the structure-function relationships in genome parts beyond the exome or in proteins difficult to characterize *in vitro*. For instance, extensive characterization of the molecular interactions of transcription factors with DNA have been limited by low-throughput methods. Application of DMS technology using NGS readout has identified functional significance of 2,700 single amino acids substitutions of the transcription factor PAX6, whose mutations cause a range of developmental disorders, with some being lethal in homozygous state (25).

Thus, increasing application of high-throughput approaches to characterize individual genomes accumulates knowledge on genetic/protein variants. Added by the knowledge on the protein structure-function relationships, that has been accumulated and conceptualized in protein science, the information is successfully applied to precise diagnostics using genetic screening against the disease gene panels, and to prediction of further gene candidates for such panels. However, complexity of the genotype-phenotype relationship must take into account a multitude of factors. For instance, the data obtained in different populations and/or ethnic groups must be compared. Populational/ethnic variety may greatly contribute to our understanding of the genotype-phenotype relationships, not the least due to different expression of consanguineous marriages in different cohorts (17, 18). Furthermore, consensus should be found on conflicting interpretation of the screening results in terms of phenotypic manifestations, which may come from different labs. To overcome these hurdles in deciphering the genotype-phenotype relationships, deposition of the data in publicly available databases is a must. Only sharing the increasingly accumulated knowledge of the sequencing results and associated clinical observations may efficiently and timely advance finding the general solution of the complex problem. The solution is absolutely necessary for accurate diagnosis of patients. At the moment, the diagnosis may take years, if at all achievable (24). In its turn, the diagnostic improvement shall bring about the higher number and better standardization of clinical descriptions of phenotypes, which are currently far behind the sequencing-generated information on genotypes. Medical doctors with a deeper basic and practical knowledge on cellular metabolism, molecular mechanisms supporting cellular homeostasis, and associated modern technologies to decipher the disease mechanisms, are required for translation of basic research and technological advances to diagnostics and therapies. Open-access publications of the case studies dealing with genotype-phenotype relationships, as well as generalizations of specific case studies and accompanying NGS data, further promote the knowledge on our understanding of genotype-phenotype relationships for elaboration of appropriate diagnostics and therapies.

In summary, modern technologies rapidly accumulate a lot of information to understand molecular mechanisms of genetically determined diseases. However, accumulation of the data *per se* does not automatically mean a better understanding of the disease origins and mechanisms. In order to translate technological advances into better diagnostics and safer disease management, the accumulated knowledge must be generalized within predictive concepts interpreting the genotype-phenotype relationship in a way, currently available for the protein structure-function relationship. These trends must be considered in programs of medical education, development of the public databases and open-access publications translating the basic knowledge to medicine.

## Author contributions

VB: Conceptualization, Writing – original draft, Writing – review & editing.

## References

[1] Sperl W Fleuren L Freisinger P Haack TB Ribes A Feichtinger RG. The spectrum of pyruvate oxidation defects in the diagnosis of mitochondrial disorders. J Inherit Metab Dis. (2015) 38:391–403. 10.1007/s10545-014-9787-3 25526709

[2] Okajima K Korotchkina LG Prasad C Rupar T Phillips JA Ficicioglu C. Mutations of the E1beta subunit gene (pdhb) in four families with pyruvate dehydrogenase deficiency. Mol Genet Metab. (2008) 93:371–80. 10.1016/j.ymgme.2007.10.135 18164639

[3] Pavlu-Pereira H Silva MJ Florindo C Sequeira S Ferreira AC Duarte S. Pyruvate dehydrogenase complex deficiency: updating the clinical, metabolic and mutational landscapes in a cohort of portuguese patients. Orphanet J Rare Dis. (2020) 15:298. 10.1186/s13023-020-01586-3 33092611 PMC7579914

[4] Singhi P De Meirleir L Lissens W Singhi S Saini AG. Pyruvate dehydrogenase-E1α deficiency presenting as recurrent demyelination: an unusual presentation and a novel mutation. JIMD Rep. (2013) 10:107–11. 10.1007/8904_2012_211 23430811 PMC3755565

[5] Narayanan RK Brewer MH Perez-Siles G Ellis M Ly C Burgess A. Charcot-marie-tooth disease causing mutation (PR158h) in pyruvate dehydrogenase kinase 3 (Pdk3) affects synaptic transmission, atp production and causes neurodegeneration in a Cmtx6 C elegans model. Hum Mol Genet. (2021) 31:133–45. 10.1093/hmg/ddab228 34387338 PMC8682796

[6] Steller J Gargus JJ Gibbs LH Hasso AN Kimonis VE. Mild phenotype in a male with pyruvate dehydrogenase complex deficiency associated with novel hemizygous in-frame duplication of the E1α subunit gene (Pdha1). Neuropediatrics. (2014) 45:56–60. 10.1055/s-0033-1341601 23572181

[7] Bedoyan JK Hecht L Zhang S Tarrant S Bergin A Demirbas D. A novel null mutation in the pyruvate dehydrogenase phosphatase catalytic subunit gene (Pdp1) causing pyruvate dehydrogenase complex deficiency. JIMD Rep. (2019) 48:26–35. 10.1002/jmd2.12054 31392110 PMC6606986

[8] Sahni N Yi S Taipale M Fuxman Bass JI Coulombe-Huntington J Yang F. Widespread macromolecular interaction perturbations in human genetic disorders. Cell. (2015) 161:647–60. 10.1016/j.cell.2015.04.013 25910212 PMC4441215

[9] Bendel AM Skendo K Klein D Shimada K Kauneckaite-Griguole K Diss G. Optimization of a deep mutational scanning workflow to improve quantification of mutation effects on protein-protein interactions. BMC Genomics. (2024) 25:630. 10.1186/s12864-024-10524-7 38914936 PMC11194945

[10] Wexler ID Hemalatha SG McConnell J Buist NR Dahl HH Berry SA. Outcome of pyruvate dehydrogenase deficiency treated with ketogenic diets. Studies in patients with identical mutations. Neurology. (1997) 49:1655–61. 10.1212/WNL.49.6.1655 9409363

[11] Koga Y Povalko N Katayama K Kakimoto N Matsuishi T Naito E. Beneficial effect of pyruvate therapy on leigh syndrome due to a novel mutation in Pdh E1α gene. Brain Dev. (2012) 34:87–91. 10.1016/j.braindev.2011.03.003 21454027

[12] Bunik V. The therapeutic potential of vitamins B1, B3 and B6 in charcot-marie-tooth disease with the compromised status of vitamin-dependent processes. Biology. (2023) 12:897. 10.3390/biology12070897 37508330 PMC10376249

[13] Artiukhov AV Aleshin VA Karlina IS Kazantsev AV Sibiryakina DA Ksenofontov AL. Phosphonate inhibitors of pyruvate dehydrogenase perturb homeostasis of amino acids and protein succinylation in the brain. Int J Mol Sci. (2022) 23:6. 10.20944/preprints202210.0006.v1 36361974 PMC9655319

[14] Pirot N Crahes M Adle-Biassette H Soares A Bucourt M Boutron A. Phenotypic and neuropathological characterization of fetal pyruvate dehydrogenase deficiency. J Neuropathol Exp Neurol. (2016) 75:227–38. 10.1093/jnen/nlv022 26865159

[15] Barnerias C Saudubray JM Touati G De Lonlay P Dulac O Ponsot G. Pyruvate dehydrogenase complex deficiency: four neurological phenotypes with differing pathogenesis. Dev Med Child Neurol. (2010) 52:e1–9. 10.1111/j.1469-8749.2009.03541.x 20002125

[16] Heebner M Mainali G Wei S Kumar A Naik S Pradhan S. Importance of genetic testing in children with generalized Epilepsy. Cureus. (2024) 16:e59991. 10.7759/cureus.59991 38854234 PMC11162283

[17] Yalcintepe S Gurkan H Dogan IG Demir S Sag SO Kabayegit ZM. The importance of multiple gene analysis for diagnosis and differential diagnosis in charcot marie tooth disease. Turk Neurosurg. (2021) 31:888–95. 10.5137/1019-5149.JTN.33661-21.3 34169998

[18] Megarbane A Bizzari S Deepthi A Sabbagh S Mansour H Chouery E. A 20-year clinical and genetic neuromuscular cohort analysis in lebanon: an international effort. J Neuromuscul Dis. (2022) 9:193–210. 10.3233/JND-210652 34602496 PMC8842757

[19] Qin D. Next-generation sequencing and its clinical application. Cancer Biol Med. (2019) 16:4–10. 10.20892/j.issn.2095-3941.2018.0055 31119042 PMC6528456

[20] Luo T Pan J Zhu Y Wang X Li K Zhao G. Association between de novo variants of nuclear-encoded mitochondrial-related genes and undiagnosed developmental disorder and autism. QJM. (2024) 117:269–76. 10.1093/qjmed/hcad249 37930872 PMC11014680

[21] Gibson GE Feldman HH Zhang S Flowers SA Luchsinger JA. Pharmacological thiamine levels as a therapeutic approach in Alzheimer's disease. Front Med (Lausanne). (2022) 9:1033272. 10.3389/fmed.2022.1033272 36275801 PMC9585656

[22] Artiukhov AV Solovjeva ON Balashova NV Sidorova OP Graf AV Bunik VI. Pharmacological doses of thiamine benefit patients with the charcot–marie–tooth neuropathy by changing thiamine diphosphate levels and affecting regulation of thiamine-dependent enzymes. Biochemistry (Moscow). (2024) 89:1161–82. 10.1134/S0006297924070010 39218016

[23] Mahdouani M Zhuri D Sezginer Guler H Hmida D Sana M Azaza M. Functional analysis of MMR gene vus from potential lynch syndrome patients. PLoS ONE. (2024) 19:e0304141. 10.1371/journal.pone.0304141 38843250 PMC11156341

[24] Ma K Gauthier LO Cheung F Huang S Lek M. High-throughput assays to assess variant effects on disease. Dis Model Mech. (2024) 17:573. 10.1242/dmm.050573 38940340 PMC11225591

[25] McDonnell AF Plech M Livesey BJ Gerasimavicius L Owen LJ Hall HN. Deep mutational scanning quantifies DNA binding and predicts clinical outcomes of Pax6 variants. Mol Syst Biol. (2024) 20:825–44. 10.1038/s44320-024-00043-8 38849565 PMC11219921

